# Perceptual inequality between two neighboring time intervals defined by sound markers: correspondence between neurophysiological and psychological data

**DOI:** 10.3389/fpsyg.2014.00937

**Published:** 2014-09-23

**Authors:** Takako Mitsudo, Yoshitaka Nakajima, Hiroshige Takeichi, Shozo Tobimatsu

**Affiliations:** ^1^Psychophysics Laboratory, Department of Informatics, Faculty of Information Science and Electrical Engineering, Kyushu UniversityFukuoka, Japan; ^2^Department of Human Science/Research Center for Applied Perceptual Science, Faculty of Design, Kyushu UniversityFukuoka, Japan; ^3^Computational Engineering Applications Unit, Advanced Center for Computing and Communication (ACCC), RIKENSaitama, Japan; ^4^Faculty of Medical Sciences, Kyushu UniversityFukuoka, Japan

**Keywords:** temporal assimilation, equality perception, N1, contingent negative variation, slow negative component

## Abstract

Brain activity related to time estimation processes in humans was analyzed using a perceptual phenomenon called auditory temporal assimilation. In a typical stimulus condition, two neighboring time intervals (T1 and T2 in this order) are perceived as equal even when the physical lengths of these time intervals are considerably different. Our previous event-related potential (ERP) study demonstrated that a slow negative component (SNCt) appears in the right-frontal brain area (around the F8 electrode) after T2, which is associated with judgment of the equality/inequality of T1 and T2. In the present study, we conducted two ERP experiments to further confirm the robustness of the SNCt. The stimulus patterns consisted of two neighboring time intervals marked by three successive tone bursts. Thirteen participants only listened to the patterns in the first session, and judged the equality/inequality of T1 and T2 in the next session. Behavioral data showed typical temporal assimilation. The ERP data revealed that three components (N1; contingent negative variation, CNV; and SNCt) emerged related to the temporal judgment. The N1 appeared in the central area, and its peak latencies corresponded to the physical timing of each marker onset. The CNV component appeared in the frontal area during T2 presentation, and its amplitude increased as a function of T1. The SNCt appeared in the right-frontal area after the presentation of T1 and T2, and its magnitude was larger for the temporal patterns causing perceptual inequality. The SNCt was also correlated with the perceptual equality/inequality of the same stimulus pattern, and continued up to about 400 ms after the end of T2. These results suggest that the SNCt can be a signature of equality/inequality judgment, which derives from the comparison of the two neighboring time intervals.

## Introduction

Cognitive time management is an essential function in human life. Adequate time estimation is necessary for our normal social functioning, such as movement, speech, and the prediction of timing (e.g., when a traffic light changes). Indeed, the time perception literature has argued that varieties of human behavior may rely on the perception of time in the seconds-to-minutes range (for reviews, see Matell and Meck, 2000; Buhusi and Meck, 2005). Interestingly, temporal judgments at sub-second timing sometimes lead to various types of perceptual distortions (i.e., illusions) in several modalities. We previously found some phenomena related to auditory and visual temporal perception in short time intervals by means of psychophysical measurements (Nakajima et al., 1991, 2014; Sasaki et al., 1998; Arao et al., 2000; Miyauchi and Nakajima, 2005, 2007; ten Hoopen et al., 2006). One is the perceptual phenomenon referred to as “temporal assimilation” (Nakajima et al., 2004; Miyauchi and Nakajima, 2007). Consider the case where three successive tone bursts (of 20 ms) are used to create two neighboring empty time intervals (T1 and T2), with durations of 120 and 200 ms, respectively. When individuals hear this type of temporal pattern, they often perceive the two intervals as almost equal, despite the physical temporal difference. This temporal assimilation occurs asymmetrically, within a range of −80 ms ≤ T1 − T2 ≤ +50 ms. Although this phenomenon is robust, its underlying neural mechanisms are poorly understood.

Here, we delineate the characteristics of human auditory temporal processing related to auditory temporal assimilation, by conducting electroencephalographic (EEG) measurement. EEG has high temporal resolution, and is suitable to extract brain responses relevant to the perception of milliseconds-to-seconds intervals, from different brain areas at the same time. Previous research in time perception has reported some event-related potential (ERP) components that could be attributed to differences in the performance of time estimation tasks (Gibbon et al., 1997; Macar and Vidal, 2003). The transient evoked component, such as N1, is known to be modulated by the parameters of stimulus properties. It has been suggested that the characteristics of these components also vary, depending on attention allocation (e.g., Lange et al., 2003; Okamoto et al., 2007; Gontier et al., 2013; Picton, 2013) to sensory signals. When one compares the lengths of two neighboring time intervals, T1 and T2, the participant might judge the lengths of two intervals by focusing on the temporal location of the tone marking the end of T1 (and simultaneously marking the beginning of T2). Therefore, the N1 latency to the second tone burst that separate the two intervals might reflect a specific allocation of temporal attention marking the separation of the two intervals.

Another ERP component is the contingent negative variation (CNV; Walter et al., 1964). Numerous studies have revealed relationships between the CNV and the processing stages of time intervals; not only in the seconds-to-minutes range, but also in the sub-second range. It is related to the memorization of time intervals (Pouthas et al., 2000; Pfeuty et al., 2003a,b), duration reproduction (Macar et al., 1999), and accumulation processes (Pouthas et al., 2000; Montfort and Pouthas, 2003; Pfeuty et al., 2003b). CNV modulation has also been observed in our previous study (Mitsudo et al., 2009). We recorded ERPs while participants were judging the equality/inequality of T1 and T2. The CNV component appeared in the frontal area during T2 presentation, and its amplitude increased as T1 was lengthened.

In addition to these components, our previous study found an ERP component that might be related to the estimation of the equality/inequality of time intervals. A slow negative component (SNCt) appeared in the right frontal areas when participants were engaged in temporal judgments, and the component was larger for stimuli that were associated with subjective inequality between T1 and T2. Equality perception, including auditory temporal assimilation, seemed to correlate with smaller SNCts.

In the present study, we examined the characteristics of these three types of ERP components, N1, CNV, and SNCt, related to temporal assimilation. By presenting time intervals marked with three successive sounds and by recording ERPs from the central and the frontal areas simultaneously, we were able to extract the brain responses in several stages of temporal judgment. We thus aimed to explore the mechanisms of human temporal perception more systematically in the present paradigm. Our particular interest was in the SNCt related to the judgment of equality/inequality to be made after T2. We compared the magnitudes of the SNCt after T2 between conditions in which equality/inequality judgments were and were not required. ERPs were recorded while participants judged the equality/inequality of two neighboring time intervals in the experimental session. ERPs were also recorded during participants' passive listening to the stimuli in the control session. We first conducted an experiment employing the same stimulus patterns as in our previous study, but with new participants. About 1 year later, we conducted another experiment with the same participants, in which the stimulus patterns were reversed in time. If the SNCt in our previous study had really reflected the brain mechanism of equality/inequality perception, it should appear in a different group of participants and for different set of stimulus patterns, and should be larger for the stimulus patterns in which the subjective inequality between T1 and T2 had been dominant, i.e., when T1 − T2 < −80 ms or T1 − T2 > 50 ms.

## Materials and methods

### Participants

Thirteen healthy volunteers with normal hearing [Mean age 20.8 (SD = 3.2) years in Experiment 1, 1 male and 12 females] participated in both Experiments 1 and 2. None of them were musically trained except in ordinary school classes. Informed consent was obtained from each participant after an explanation of the purpose and procedures of the experiment, which were approved by the Ethics Committee of the Graduate School of Medical Sciences, Kyushu University.

### Apparatus and stimuli

**Figure 1:**
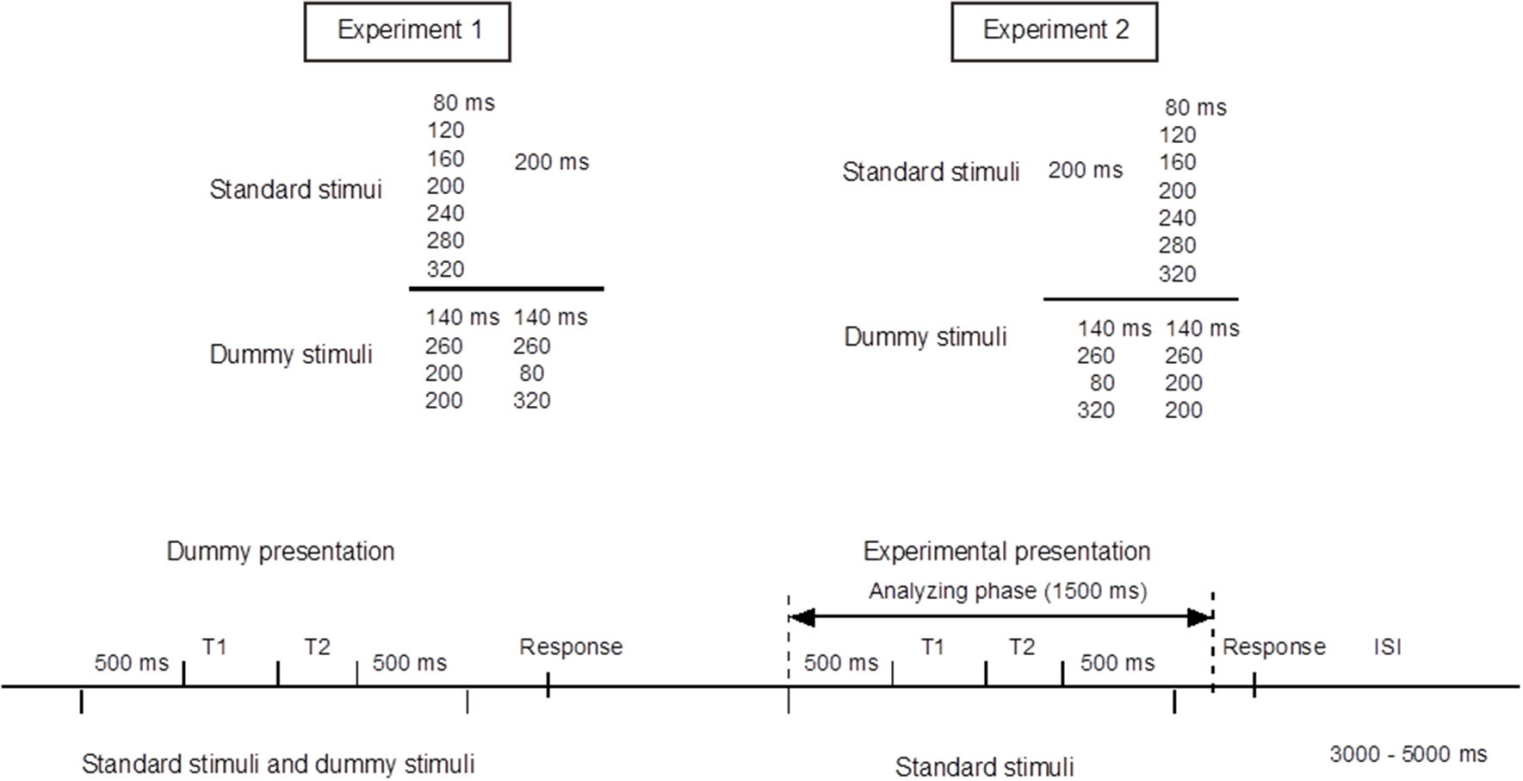
**Upper panel:** Standard and dummy stimuli in Experiments 1 and 2. **Bottom panel:** Experimental procedures. Stimulus epochs began 500 ms prior to the stimulus onset, and continued 1000 ms after the stimulus onset. Inter-stimulus intervals (ISIs) were randomly varied between 3 and 5 s. The procedures of Experiments 1 and 2 were exactly the same except that the temporal patterns were reversed in time.

The experiments were conducted in an electromagnetically shielded soundproof room (Yamaha Music Cabin, SC-3 or SC-5). The background noise was kept below 30 dBA. Stimuli were synthesized with J software (with a sampling frequency of 44.1 kHz) run on a Dell Dimension 4500C personal computer. They were presented diotically from an AV tachistoscope (Iwatsu, IS-703) via a low-pass filter (NF DV8FL with a cutoff frequency of 8 kHz), an amplifier (Stax SRM-313), and headphones (Stax SR-303, STAX). All stimulus patterns consisted of two neighboring time intervals marked by three successive pure-tone bursts of 1 kHz and 20 ms with rise and fall times of 5 ms. We labeled the three markers S1, S2, and S3. The sound pressure level of these tone bursts was 77 dBA. This level was measured as the level of a continuous tone of the same amplitude with a precision sound level meter (Node 2075), mounted on an artificial ear (Brüel and Kjær 4153). In Experiment 1, we used seven standard stimulus patterns, in which T1, defined as the inter-onset interval between the first and second marker, varied from 80 to 320 ms in 40-ms steps, whereas T2, the inter-onset interval between the second and third marker, was fixed at 200 ms. In Experiment 2, the same apparatus was used, and the stimulus patterns were reversed in time; T1 was fixed at 200 ms, and T2 varied from 80 to 320 ms. In each of these experiments, we used four dummy stimulus patterns to prevent the participants from memorizing or noticing the fixed 200-ms duration. Indicating the neighboring time intervals as T1|T2 ms, the dummy patterns were 140|140, 260|260, 200|80, and 200|320 ms in Experiment 1, and the same patterns were reversed in time in Experiment 2 (Figure 1).

### Procedures

We first conducted Experiment 1. Experiment 2 was conducted about 1 year later with the same participants. Each experiment consisted of an experimental session and a control session. The task in the experimental session was to judge whether the durations of T1 and T2 were equal or unequal by pressing one of the two buttons on a unit held with both hands. The task in the control session was to listen passively to the stimuli and to press one of the two buttons, chosen at the participant's own will, without making a judgment. For both the experimental and the control sessions, the seven standard stimuli and four dummy stimuli were presented. One trial started with a dummy presentation followed by an experimental presentation. The combination of stimuli presented in the dummy presentation and the experimental presentation was randomized. Each of the seven standard stimuli were presented 100 times in random order in the experimental presentations, while the dummy presentations, in which the dummy and the standard stimuli were employed, alternated with the experimental presentations to avoid participants memorizing or noticing the fixed intervals in the standard stimuli (Figure 1). The sessions were divided into 10 blocks of 40 trials and 10 blocks of 30 trials (i.e., 7 standard stimuli × 10 blocks × 10 trials). ERPs were recorded only in the experimental presentations. Inter-stimulus intervals (ISIs) were varied randomly between 3 and 5 s. Each participant first performed the control session and then the experimental session on four separate days in total.

### ERP recordings

ERPs were recorded from 19 scalp locations (Fp1, Fp2, F7, F8, Fz, F3, F4, Cz, C3, C4, Pz, P3, P4, T3, T4, T5, T6, O1, and O2; international 10-20 system) referred to an electrode at the nose tip, using EEG-1100 (Neurofax, Nihon Koden). Horizontal and vertical electro-oculograms (EOGs) were also recorded using four electrodes placed over the outer canthi and in the superior and inferior areas of the orbit. The electrode impedance was kept below 5 kΩ. The ERP and EOG data were band-pass filtered between 0.27 and 300 Hz, and sampled at a rate of 683 Hz. For the ERP analysis, each stimulus epoch began 500 ms prior to, and continued 1000 ms after, the onset of the first marker (Figure 1). The participant was instructed to close their eyes and yet to stay alert. Trials that included artifacts, defined as waves for which voltage exceeded ±100 μV at one or more electrodes, were excluded from the analyses.

## Results

### Behavioral data

**Figure 2:**
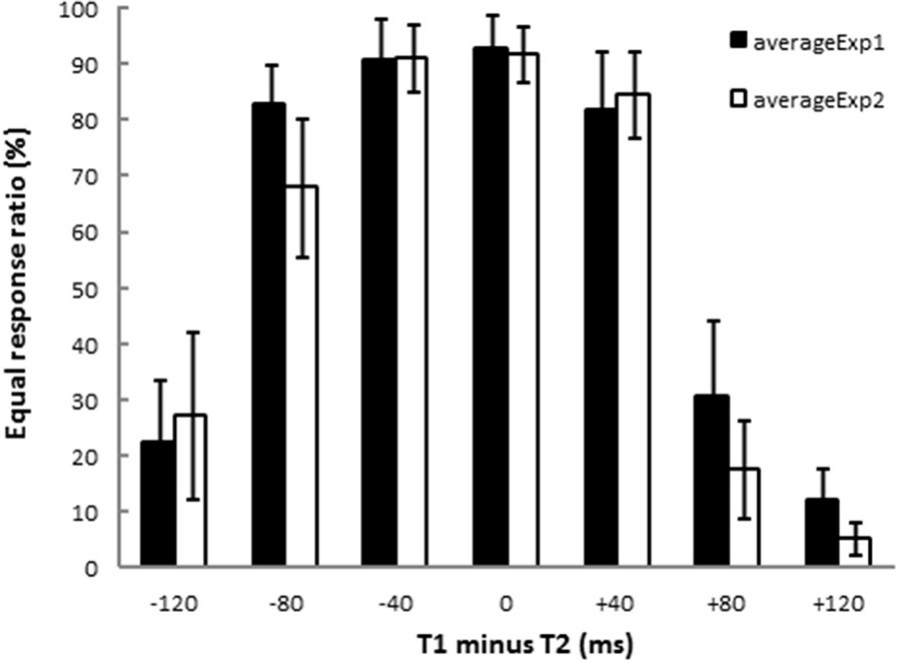
**Results of equal/unequal judgments**. Each bar shows the ratio of equal responses (i.e., T1 and T2 were perceived as having the same duration). The black and the white bars show the results of Experiment 1 and Experiment 2, respectively. T1 and T2 were perceived as equal when −80 ≤ (T1 − T2) ≤ +40 ms. The results indicate that asymmetrical temporal assimilation took place between T1 and T2 in both experiments.

Figure 2 shows the results of the equal/unequal judgments. We assessed the equal response ratio defined as the proportion of trials in which participants judged the two time intervals as equal. After an inverse sine transformation, response ratios in Experiments 1 and 2 were subjected to ANOVA (T1 − T2: −120, −80, −40, 0, +40, +80, +120 ms)^1^. There were significant main effects of T1 − T2 [Experiment 1: *F*_(6, 84)_ = 46.89, *p* < 0.001, η^2^_*p*_ = 0.76, and Experiment 2: *F*_(6, 84)_ = 44.60, *p* < 0.001, η^2^_*p*_ = 0.75]. Dunnett's *post-hoc t*-test was performed for each experiment to check whether the equal response ratios obtained from 6 stimulus patterns (T1 − T2 = −120, −80, −40, +40, +80, +120 ms) differed from that for the stimulus pattern of physically equal time intervals (T1 − T2 = 0 ms). The response ratios differed significantly from that obtained for T1 − T2 = 0 ms when T1 − T2 was −120, +80, or +120 ms both in Experiment 1 (200|200 vs. 80|200: *p* < 0.001, 200|200 vs. 280|200: *p* < 0.001, 200|200 vs. 320|200: *p* < 0.001) and in Experiment 2 (200|200 vs. 200|80: *p* < 0.001, 200|200 vs. 200|120: *p* < 0.001, 200|200 vs. 200|320: *p* < 0.001). In both experiments, T1 was perceived as equal to T2 when the difference between T1 and T2, T1 − T2, was in an asymmetrical range from −80 to 40 ms. The asymmetrical temporal assimilation indeed occurred.

### ERP data

**Figure 3:**
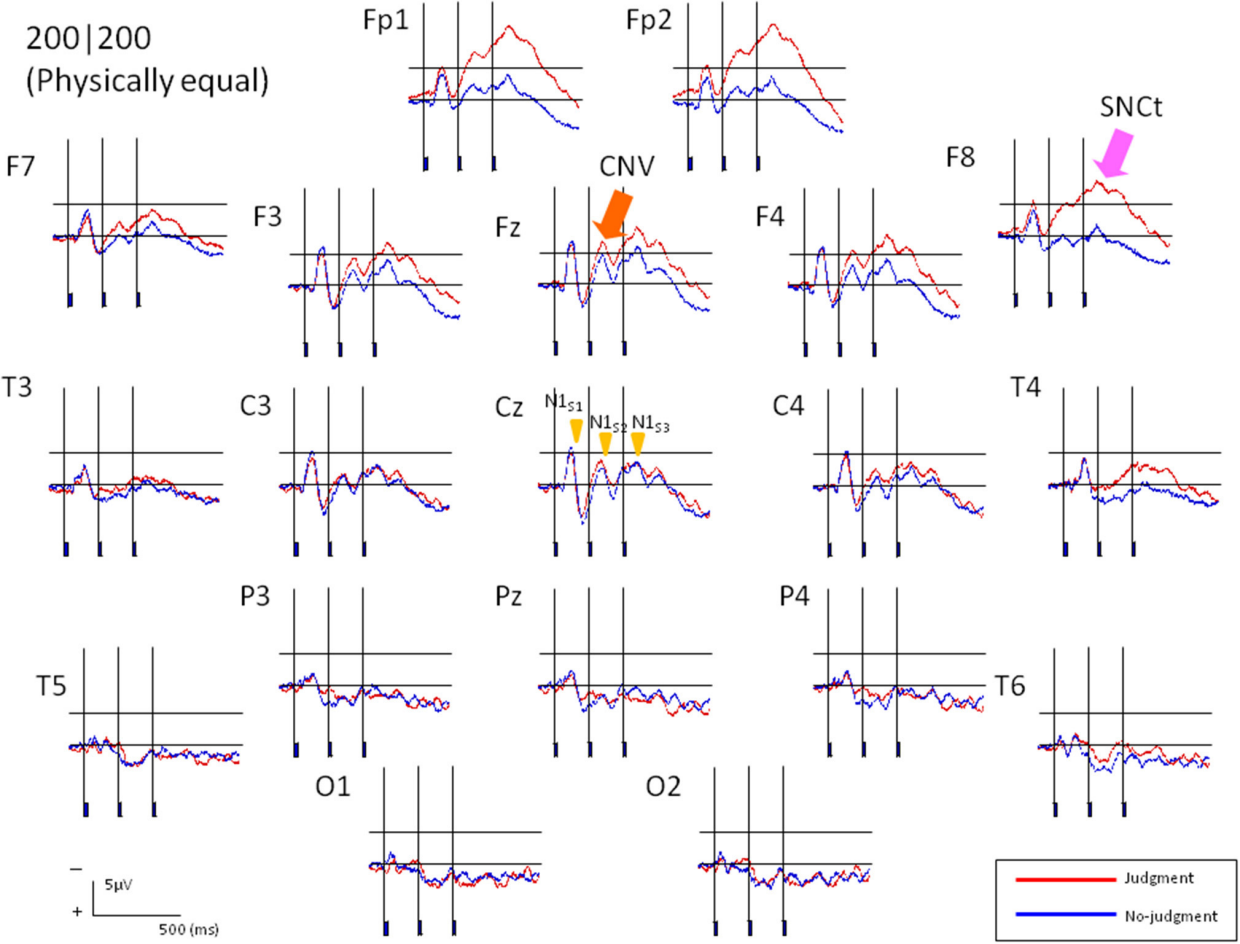
**Averaged ERP responses of 13 participants elicited in the condition where T1 = T2 = 200 ms (physically equal) in Experiment 1**. The two neighboring time intervals were perceived as equal. Red lines represent the ERPs obtained when the participants made equal/unequal judgments, while the blue lines indicate the control task, in which they listened to the stimuli passively. N1 peak latencies were clearly observed at Cz for both experimental and control tasks. The CNV emerged in the frontal areas and the SNCt emerged in the right-frontal areas.

ERPs were obtained by averaging the EEG waveforms for each of the seven stimulus patterns. Figure 3 shows grand averaged ERP waveforms of 13 participants elicited in the condition where T1 = T2 = 200 ms in Experiment 1. N1 appeared maximally at the central area (Cz). A CNV-like component appeared at the frontal area (Fz) during the stimulus presentation. The SNCt emerged in the right-frontal area at approximately 300 ms after the first marker and lasted until 400 ms after the third marker. The SNCt amplitudes in the experimental session were greater in the right-frontal areas than those in the left corresponding areas. These components were observed in all stimulus patterns and in Experiment 2 as well.

#### N1

**Figure 4:**
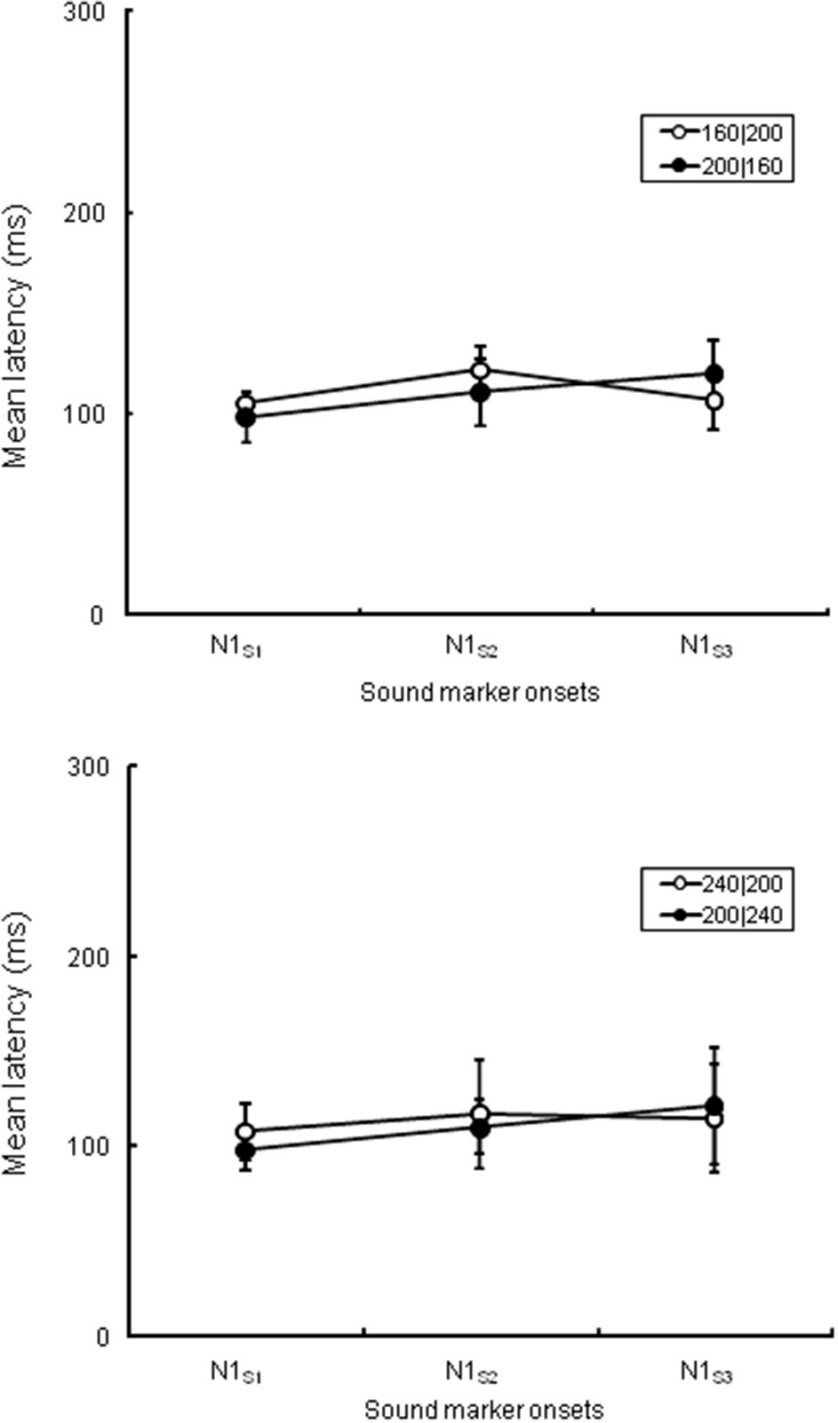
**N1 peak latencies of each sound marker (N1_S1_, N1_S2_, and N1_S3_, respectively) measured from the stimulus onset at Cz in the experimental session**. The upper graph is comparison of peak latencies of N1_S1_, N1_S2_, and N1_S3_ between 160|200 in Experiment 1 and 200|160 in Experiment 2, while the lower graph between 240|200 in Experiment 1 and 200|240 in Experiment 2. All stimulus patterns were perceived as nearly equal in behavioral data even though the lengths of T1 and T2 were physically different.

First, we analyzed the ERP components at the central electrode (Cz) to check the spatiotemporal characteristics of the ERP components related to the temporal judgment. We focused on a transient negative component (N1) at Cz, corresponding to the three sound markers' onsets (N1_S1_, N1_S2_, and N1_S3_), and checked the attentional effects on the N1_S2_ that separated the two intervals. In the analysis, all sound marker onsets were located relative to the timing of each marker onset. We selected mirror-pairs of stimulus patterns where T1 and T2 were perceived as nearly equal in the behavioral results (see Figure 2), even though the physical durations of these intervals were different (i.e., 160|200, 200|160, and 240|200, 200|240). The differences of N1 peak latency to each of the three sound markers in the experimental session were measured from the baseline. A 3 (N1: N1_S1_, N1_S2_, and N1_S3_) × 2 (Experiment: 1 and 2) ANOVA with repeated measures was performed for each pair of stimulus patterns to check whether or not N1 latencies varied related to equality perception. The Greenhouse-Geisser correction was applied to the ANOVA when the sphericity assumption was violated in the dependent measures. The Bonferroni correction for multiple post-hoc comparisons was applied when required. The η^2^_*p*_ (partial eta-squares) were calculated for the quantitative comparison of effect sizes. Given that the N1 latency to each sound marker reflects attentional effects on the sensory signals, the N1 peak might be shifted to the temporal point where the two time intervals are assimilated. Table 1 and Figure 4 show the means (and SDs) of the peak N1 latencies for N1_S1_, N1_S2_, and N1_S3_ of each stimulus pattern in the experimental session, measured relative to each marker onset. For the mirror-pair of 160|200 and 200|160, there was a main effect of N1 [*F*_(2, 24)_ = 6.26, *p* < 0.01, η^2^_*p*_ = 0.34]. The effect of Experiment [*F*_(1, 12)_ = 0.13, *n.s*., η^2^_*p*_ = 0.01] and the interaction of N1 and Experiment [*F*_(2, 24)_ = 2.33, *n.s*., η^2^_*p*_ = 0.15] were not significant. Peak latencies for N1_S2_ and N1_S3_ extended gradually, and the differences in N1 peak latencies for each mirror-pair did not appear. For the mirror-pair of 240|200 and 200|240, the main effects of N1 [*F*_(2, 24)_ = 3.93, *p* < 0.05, η^2^_*p*_ = 0.25] was significant. Neither the main effects of Experiment [*F*_(1, 12)_ = 0.34, *n.s*., η^2^_*p*_ = 0.03] nor the interaction of N1 and Experiment [*F*_(2, 24)_ = 1.78, *n.s*., η^2^_*p*_ = 0.12] were significant. As in former patterns, the differences of N1 peak latencies for each mirror-pair did not appear.

**Table 1:** **N1_S1_, N1_S2_, and N1_S3_ latencies of each stimulus pattern**.

**Component**	**Stimulus pattern [ms | ms]**	**Mean latency (SD) [ms]**
N1_S1_	160|200	104.0 (12.3)
	200|160	97.1 (12.2)
N1_S2_	160|200	120.5 (24.4)
	200|160	109.5 (16.4)
N1_S3_	160|200	105.2 (27.1)
	200|160	118.6 (16.4)
N1_S1_	240|200	107.8 (15.5)
	200|240	98.0 (10.0)
N1_S2_	240|200	117.3 (28.7)
	200|240	109.8 (14.5)
N1_S3_	240|200	114.5 (28.8)
	200|240	121.3 (19.3)

#### CNV

**Figure 5:**
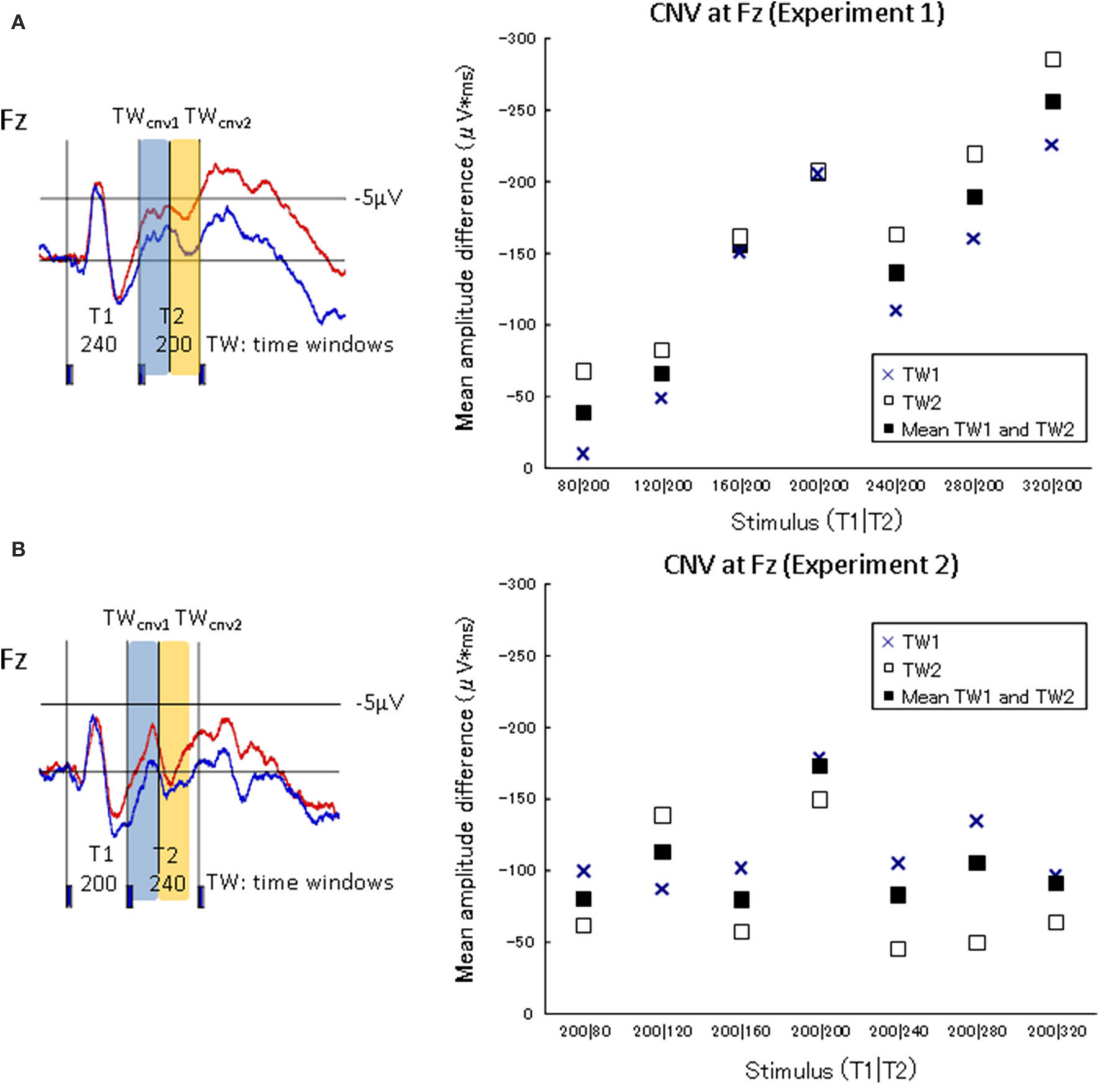
**CNV amplitude differences between the judgment condition and the no-judgment condition in TWcnv_1_ and TWcnv_2_**. The upper graph shows the results of Experiment 1, while the lower graph of Experiment 2. The waveforms **A** and **B** are indicated to specify the temporal ranges of TW_CNV1_ and TW_CNV2_ in Experiments 1 (T1|T2 = 240|200) and 2 (T1|T2 = 200|240), respectively. In Experiment 1, the CNV amplitudes in the experimental task increased as T1 was lengthened.

The characteristics of neural activity during stimulus presentation were examined. The CNV difference waves at the frontal electrode (Fz) in the experimental session and the control session were calculated over two successive 100-ms time windows (TWcnv) from the onset of the second marker to the onset of the third marker (i.e., T2) of Experiment 1. The corresponding TWs in Experiment 2 were also calculated (i.e., from the onset of the second marker to the time windows 200 ms after the second marker)^2^. The CNV difference waves at Fz in each of the seven standard stimuli in Experiment 1 (80|200, 120|200, 160|200, 200|200, 240|200, 280|200, and 320|200) and Experiment 2 (200|80, 200|120, 200|160, 200|200, 200|240, 200|280, and 200|320) were integrated within each TWcnv on Fz for each participant. The CNV difference waves in the frontal area should increase as T1 was lengthened in Experiment 1, if they accompanied the process of memorizing the lengths of T1. Figure 5 shows the amplitude differences of the CNVs between the experimental session and the control session in TWcnv_1_ and TWcnv_2_. The CNV difference waves of TWcnv_1_ and TWcnv_2_ in Experiment 1 were fitted to a linear regression curve. Adjusted R-squared and Spearman's ρ were calculated to check whether the CNV amplitude differences in TWcnv_1_ and TWcnv_2_ changed as a function of preceding time intervals (i.e., T1). The length of T1 and the averaged CNV differences for TWcnv_1_ and TWcnv_2_ were positively correlated (TWcnv_1_:*R*^2^ = 0.58, TWcnv_2_:*R*^2^ = 0.84) and significant (TWcnv_1_:Spearman's ρ = 0.82, *p* < 0.02, TWcnv_2_: Spearman's ρ = 1.00, *p* < 0.01). In Experiment 2, the CNV difference waves of TWcnv_1_ and TWcnv_2_ did not change, as expected from the present results of Experiment 1 and our previous results (Mitsudo et al., 2009; Experiment 2).

#### SNCt

We focused on six frontal electrodes (Fp1, Fp2, F7, F8, F3, and F4) where a post-stimulus SNCt emerged. Because any temporal comparison must have taken place only after the participant had a chance to perceive both of the neighboring time intervals, the ERPs corresponding to the judgment were expected to appear after the third marker (Paul et al., 2011). To examine the SNCt, the stimulus epoch up to 400 ms after the end of the third marker was divided into four time windows (TW_SNCt_s) of 100 ms: TW_SNCt1_ to TW_SNCt4_. We calculated the SNCt difference waves by subtracting the mean SNCt amplitudes in the control session from those in the experimental session. The SNCt difference waves were integrated within each TW_SNCt_ on all of the 19 scalp electrodes. The integrated values of six frontal electrodes (Fp1, Fp2, F7, F8, F3 and F4) were used for further statistical analyses. The means (SDs) of the SNCt difference waves are shown in Table 2.

**Table 2:** **The means (SDs) of the SNCt difference waves categorized by the equal-dominant and the unequal-dominant stimulus patterns at left (Fp1, F7, and F3) and right (Fp2, F8, and F4) frontal electrodes**.

**Time Window [ms]**	**0–100**		**100–200**		**200–300**		**300–400**	
**Behavioral response**	**Equal**	**Unequal**	**Equal**	**Unequal**	**Equal**	**Unequal**	**Equal**	**Unequal**
Experiment 1 Left	281.5 (390.4)	353.5 (453.8)	330.3 (422.1)	387.4 (482.7)	254.6 (350.9)	358.9 (418.5)	213.1 (402.7)	340.0 (396.4)
Right	218.6 (318.1)	407.1 (436.7)	255.4 (335.5)	448.5 (460.0)	239.7 (330.7)	405.1 (444.9)	185.6 (358.3)	386.6 (458.0)
Experiment 2 Left	37.4 (187.2)	140.6 (427.6)	62.5 (271.3)	203.9 (558.2)	−15.7 (269.5)	176.6 (631.1)	−27.5 (287.5)	120.7 (577.6)
Right	140.9 (304.4)	215.8 (427.2)	196.8 (363.9)	279.1 (541.8)	136.3 (374.2)	264.5 (592.4)	134.3 (385.8)	230.8 (583.5)

*Values are in μV*.

**Figure 6:**
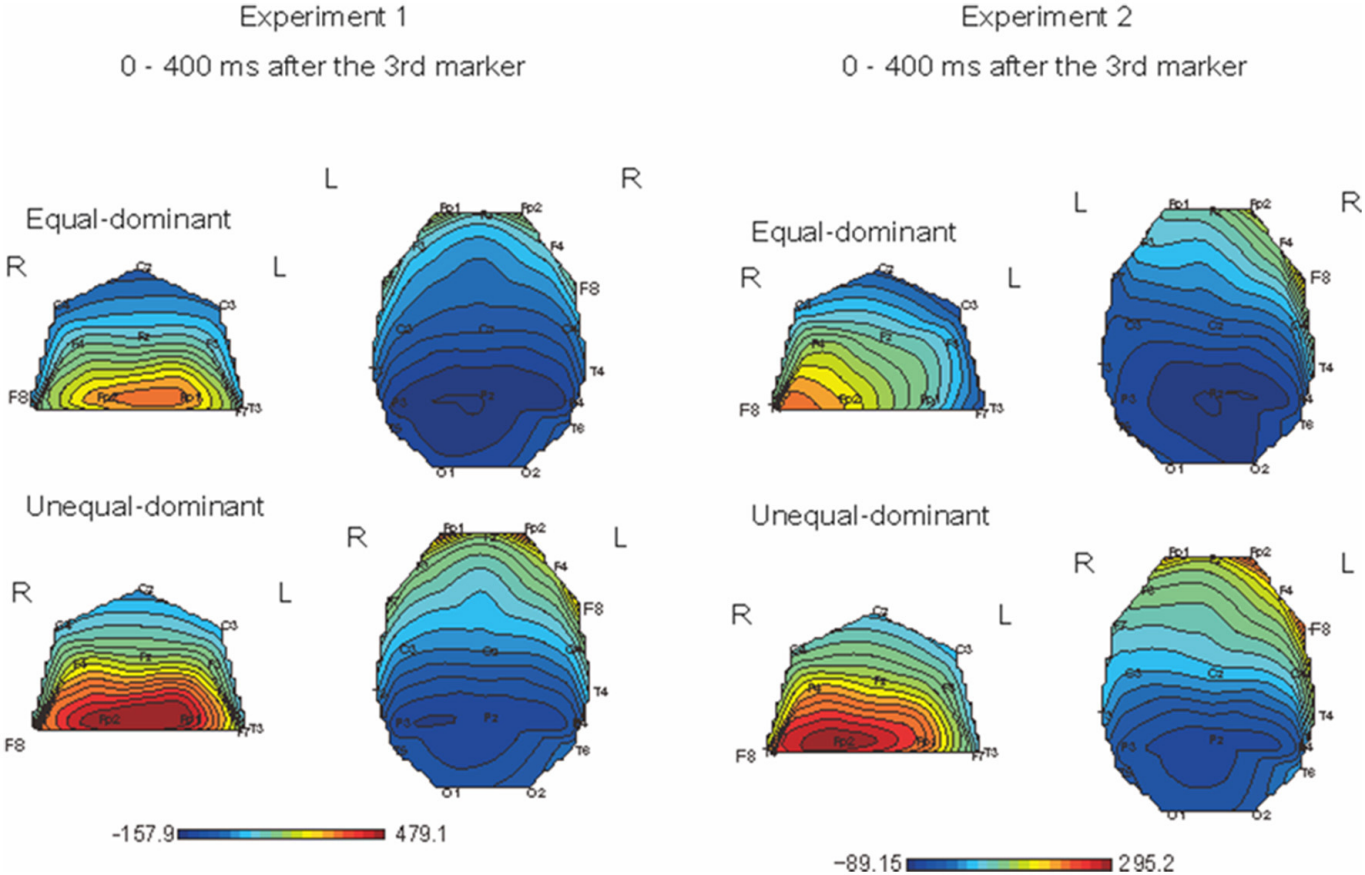
**Topographical mapping of brain activity during equal and unequal judgments of the standard stimuli (Left: Experiment 1, Right: Experiment 2)**. The maps show the brain activity in the time windows within 400 ms after the third marker. In both Experiments 1 and 2, the right-frontal areas' activation was significantly larger for the unequal-dominant stimulus patterns (i.e., T1 − T2 = −120, +80, +120 ms) than for the equal-dominant stimulus patterns (i.e., T1 − T2 = −80, −40, 0, +40 ms). In Experiment 2, the SNCt derived from the right-frontal electrodes was significantly larger than that from the left.

We first divided the ERPs into two groups: those obtained in the conditions where equal judgments dominated (i.e., T1 − T2 = −80, −40, 0, +40 ms) and those obtained in the conditions where unequal judgments dominated (i.e., T1 − T2 = −120, +80, +120 ms). Figure 6 shows the color maps of the brain activity corresponding to equal- and unequal-dominant stimulus patterns in Experiment 1 (Figure 6, left figure) and Experiment 2 (Figure 6, right figure), in which the SNCt difference waves up to 400 ms after the third marker were averaged. A remarkable difference between these two groups was observed in the frontal area. A three-way (4 time windows (TWs) × 2 laterality × 2 equality) repeated-measures ANOVA was performed over left- (Fp1, F7, and F3) and right- (Fp2, F8, and F4) frontal electrodes, to check for the effects of laterality and equal/unequal judgment in each TW. In Experiment 1, the main effect of equal/unequal judgment was significant [*F*_(1, 12)_ = 5.95, *p* = 0.03, η^2^_*p*_ = 0.33]. Multiple comparisons with the Bonferroni correction revealed that the SNCt in the unequal-dominant stimulus patterns was significantly larger than that in the equal-dominant stimulus patterns (equal vs. unequal: *p* = 0.03). The effect of laterality was not significant [*F*_(1, 12)_ = 0.003, *n.s*., η^2^_*p*_ = 0.00]. The interaction between equal/unequal judgment and laterality was significant [*F*_(1, 12)_ = 4.90, *p* = 0.04, η^2^_*p*_ = 0.29]. Multiple comparisons showed that the SNCt in the right-frontal area was larger in unequal-dominant stimulus patterns than in equal-dominant stimulus patterns (equal vs. unequal: *p* = 0.011). In Experiment 2, the main effect of laterality was significant [*F*_(1, 12)_ = 10.38, *p* = 0.007, η^2^_*p*_ = 0.46]. The SNCt in the right frontal area was significantly larger than that in the left (right vs. left: *p* = 0.007). The effect of equality was not significant [*F*_(1, 12)_ = 0.49, *n.s*., η^2^_*p*_ = 0.04]. The interaction between laterality and TWs was significant [*F*_(1.53, 1.84)_ = 4.61, *p* = 0.03, η^2^_*p*_ = 0.28]. Multiple comparisons indicated that the neural activity derived from the right-frontal electrodes was larger than that derived from the left-frontal electrodes between 0 to 400 ms after the onset of the third marker (TW1: *p* = 0.02, TW2: *p* = 0.008, TW3: *p* = 0.009, and TW4: *p* = 0.004).

To investigate the relationship between the SNCt and the judged equality/inequality, we performed a new type of selective averaging of the ERP data. Trials in which participants responded “equal” or “unequal” were averaged separately. We took the data obtained when T1|T2 = 280|200 and 200|280 for the following reasons. First, in these patterns, the temporal differences between T1 and T2 were both physically 80 ms. Second, behavioral data showed that the perception for these temporal patterns had some ambiguity: these patterns caused both “equal” and “unequal” judgments to substantial amounts, although the “unequal” judgment dominated for 280|200, while the “equal” judgment dominated for 200|280. Waveforms of T1|T2 = 280|200 and 200|280 were divided and averaged selectively in terms of “equal” and “unequal” responses. The SNCt difference waves were calculated by subtracting the mean SNCt amplitudes in “equal” responses from those in “unequal” responses. As in the stimulus-based analysis, the SNCt difference waves were integrated within each TW_SNCt_ on all of the 19 scalp electrodes, and the integrated values of 6 frontal electrodes (left: Fp1, F7, and F3, and right: Fp2, F8, and F4) were used for the analyses.

**Figure 7:**
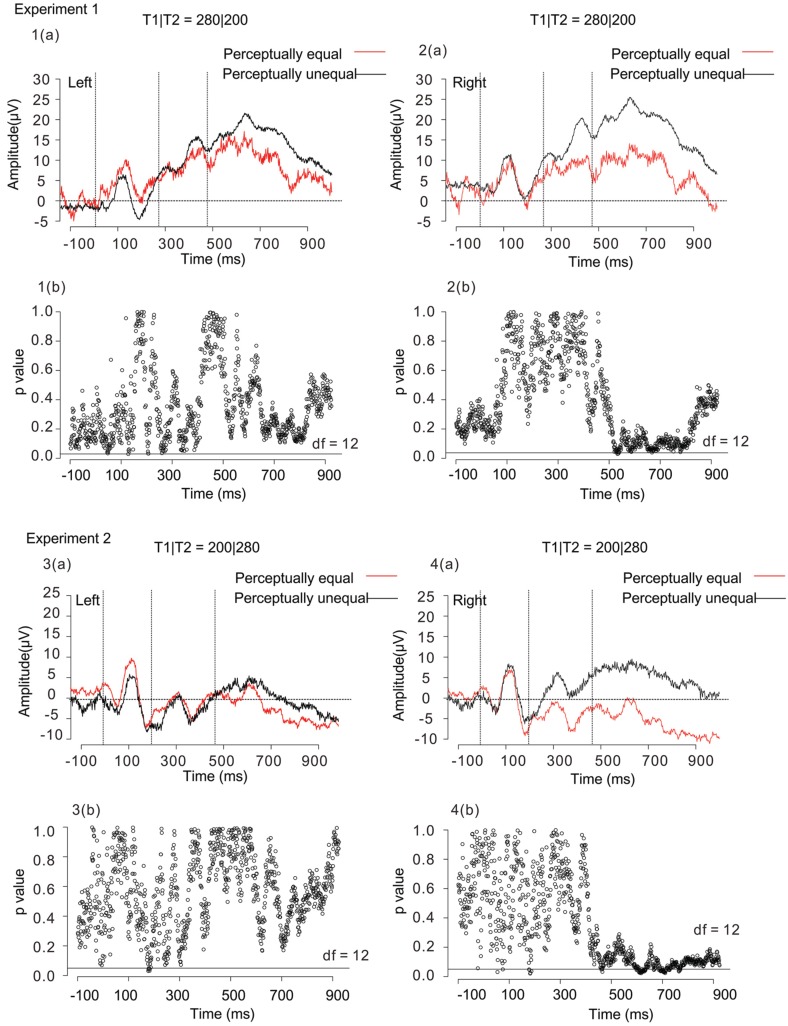
**Averaged waveforms of 13 participants obtained from 3 right-frontal (Fp2, F8, and F4) and 3 left-frontal (Fp1, F7, and F3) electrodes in Experiment 1 [1(a) and 2(a)] and Experiment 2 [3(a) and 4(a)]**. Black lines represent the ERPs for participants' unequal perception, while red lines those for participants' equal perception. The figures below each waveform are the results of paired *t*-tests (*p* < 0.05, two-tailed) between “equal” and “unequal” response-based averaging waveforms [1(b), 2(b), 3(b), and 4(b)]. The time interval of significance was identified when a paired *t*-test reached significance. The open circles in each figure represent *p*-values in terms of the comparison between “equal” and “unequal” waveforms at each time point. The horizontal black lines in the figure represent the significance level (*p* < 0.05). Both in Experiment 1 and Experiment 2, the SNCt in the right-frontal electrodes was large when participants judged two time intervals as subjectively “unequal.” The ERP differences between “equal” and “unequal” perceptions started within 80 ms after the stimulus in T1|T2 = 280|200 (an unequal-dominant stimulus pattern), while immediately after the third marker in T1|T2 = 200|280 (an equal-dominant stimulus pattern) in Experiment 2.

Figure 7 shows response-based selective averaging ERPs of T1|T2 = 280|200 and 200|280, obtained from three frontal electrodes of right (Fp2, F8, and F4) and left (Fp1, F7, and F3) in Experiments 1 and 2 [Figure 7, 1(a)–4(a)]. “Unequal” responses were averaged: 531 trials in Experiment 1, and 424 trials in Experiment 2. “Equal” responses were averaged as well: 293 trials in Experiment 1, and 639 trials in Experiment 2. We compared the averaged waveforms of the left and right SNCt between “equal” and “unequal” judgments of T1|T2 = 280|200 and 200|280, by conducting a paired *t*-test at each time point. The figures below each waveform are the results of *p*-values from the paired *t*-test (*df* = 12, *p* < 0.05) between “equal” and “unequal” response-based averaging [Figure 7, 1(b)–4(b)]. The results of the paired *t*-test showed that the averaged waveforms in the right-frontal electrodes were large when participants judged two time intervals as subjectively “unequal” both in Experiments 1 and 2. This tendency was observed both in 280|200 (an unequal-dominant stimulus pattern) and 200|280 (an equal-dominant stimulus pattern). The ERP differences between “equal” and “unequal” judgments started at 520 ms, approximately 40 ms after the third marker in Experiment 1 and at 480 ms, immediately after the third marker in Experiment 2, corresponding to the SNCt.

We further conducted a two-way (4 time windows (TWs) × 2 laterality) repeated-measures ANOVA for each of T1|T2 = 280|200 and 200|280, to check for effects of laterality in each TW. For both in Experiments 1 and 2, the main effects did not reach significance either in the time windows [Experiment 1: *F*_(1.26, 15.2)_ = 0.42, *n.s*., η^2^_*p*_ = 0.03, and Experiment 2: *F*_(1.8, 21.5)_ = 0.41, *n.s*., η^2^_*p*_ = 0.03], or in the laterality [Experiment 1: *F*_(1, 12)_ = 0.59, *n.s*., η^2^_*p*_ = 0.05, and Experiment 2: *F*_(1, 12)_ = 3.2, *n.s*., η^2^_*p*_ = 0.21].

## Discussion

The purpose of this study was to examine the characteristics of three types of ERP components, N1, CNV, and SNCt, which can be associated with temporal equality/inequality. Behavioral results showed that assimilation took place in an asymmetrical time range of −80 ≤ (T1 − T2) ≤ +50 ms. This result agrees with previous psychophysical findings (Nakajima et al., 2004; Miyauchi and Nakajima, 2007; Mitsudo et al., 2009), and demonstrates the robustness of this asymmetrical tendency. The N1 appeared corresponding to the physical onset of each marker, and the CNV appeared as a function of T1. These two components appeared during the presentation of the stimuli. The SNCt appeared at the right-frontal brain area 0–400 ms after the third marker, also for the new stimulus patterns in Experiment 2. The reproducibility suggests that this component is related to the equality/inequality perception of time intervals. Our results also show that the analysis of several different ERP indices is necessary to understand temporal processing, from stimulus detection to decision making related to the equality/inequality perception.

### Auditory evoked onset responses corresponding to the three temporal markers

Peak latencies of N1 in response to the sound markers appeared corresponding to the physical timing of each marker onset (Figure 4 and Table 1). The differences in N1 peak latencies between two mirror-pairs for each sound marker, N1_S2_, N1_S1_, and N1_S3_, did not exhibit the statistical differences between the pairs of stimulus patterns. The N1 peak latencies to each sound marker appeared constantly at approximately 100 or 110 ms after stimulus onset. The specific allocation of temporal attention would not reflect on the timing of these sensory signals. This is somewhat against of previous studies reporting the enhancement of N1 amplitude (e.g., Hillyard et al., 1973; Lange et al., 2003) and the shortening of N1 latency (Okamoto et al., 2007) caused by attention—a recent report has suggested that the N1 is more closely related to the temporal structures of stimulus patterns than the CNV (Kononowicz and van Rijn, 2014). In our study, N1 responses that separated the intervals did not exhibit any latency shortening related to temporal judgment. The N1 in the present case must have been related to neural activity that was time-locked to the onsets of the stimuli, but not to the attention that affects equality/inequality judgment.

### The CNV activity observed over the frontal site after the second marker

In Experiment 1, the CNV amplitudes in the experimental task increased as T1 was lengthened. In Experiment 2, in which T1 was always fixed at 200 ms, the CNV kept the same amplitude during the same temporal windows as in Experiment 1 (Figure 5). When the first interval (T1) was varied from 80 to 320 ms in Experiment 1, the information regarding the duration of T1 was probably retained in subsequent time windows to compare it with the second interval (T2). Thus, the memorized duration of T1 was reflected to the EEG changes in TW1 and TW2, and the CNV amplitudes increased linearly as a function of T1. In contrast, when the first intervals were fixed (at 200 ms in the current study) in Experiment 2, the preserved information of T1 should have been constant across all the stimulus conditions. This explains the fact that the CNV amplitudes did not change. Previous ERP studies adopting temporal judgment tasks have reported that the CNV amplitudes became larger when perceived time length was estimated as longer (Pfeuty et al., 2003b; Le Dantec et al., 2007; Mitsudo et al., 2012; Gontier et al., 2013). The CNV, which was defined as the brain activity up to 200 ms after the onset of the second marker in the present study, must have changed depending on the memorized duration of the intervals. The CNV observed over the frontal site was assumed to be related to the memorization of time intervals, which is in line with previous studies where the CNV amplitudes were larger when subjectively judged time was longer (Macar et al., 1999; Pfeuty et al., 2003a,b; Le Dantec et al., 2007; but see different views; Kononowicz and van Rijn, 2011, 2014; van Rijn et al., 2011).

### Brain activity derived from equal-dominant/unequal-dominant stimulus patterns

The SNCt emerged most prominently around the right-frontal electrodes. The SNCt observed in our previous study appeared clearly in a new set of data recorded from different participants for increased stimulus patterns. When the ERPs were divided for equal-dominant and unequal-dominant stimulus patterns, the SNCt derived from the right-frontal brain area was larger in the unequal-dominant stimulus patterns (Figure 6). Previous studies that examined the EEG signatures of temporal discrimination argued that the decision processes in temporal discrimination were reflected in a component that appeared after the presentation of both durations to be compared (Gontier et al., 2009; Paul et al., 2011). The SNCt, which involved brain activities after T1 and T2, was considered as an index of decision processes regarding the subjective temporal judgments. This is in accord with the notion that the right-frontal brain area plays a crucial role in the perception of time (Pfeuty et al., 2003a,b; Rubia and Smith, 2004; Hairston and Nagarajan, 2007).

### Neural correlates of perceptual equality/inequality

The most interesting finding in the current study was that the SNCts at the right-frontal electrodes showed larger activities when the brain processed perceptual inequality, rather than equality, of the two neighboring time intervals, which was revealed in T1|T2 = 280|200 and 200|280 (Figure 7). Even if participants listened to the same stimulus pattern, the SNCt in the right-frontal electrodes was different when they judged two neighboring time intervals as “equal” or “unequal.” This tendency was observed both in 280|200 (an unequal-dominant stimulus pattern) and 200|280 (an equal-dominant stimulus pattern). The magnitude of the SNCt changed corresponding to the behavioral responses even to physically identical stimulus patterns. Previous studies reported that the slow positive component that is considered to be related to the decision processes appeared in the prefrontal cortex during duration discrimination and it emerged within 500 ms after the stimulus offset (Gontier et al., 2009; Paul et al., 2011); it is very likely that the SNCt was also related to the decision processes regarding the equality/inequality of two neighboring time intervals.

Earlier studies have documented that the brain attenuates its activities when a temporal task is performed more efficiently (Casini and Macar, 1996). The magnitudes of SNCt can be connected to the economic information processing in the brain (Nakajima et al., 2004). When the successively presented sounds are assumed to create regular time intervals, the brain is probably able to save its activity. This may result in the low SNCt amplitude at the right-frontal areas in the equal responses.

According to a psychophysical model of unilateral temporal assimilation (Nakajima et al., 2004), the perceived difference between T1 and T2 could be reduced by cutting the processing time for T2 after the offset of the third marker. If this model works in the present experimental paradigm, the whole of the processing, including the detection of the markers, basically continues about 80 ms after the third marker's onset. In T1|T2 = 200|280, in which unilateral temporal assimilation (time-shrinking) probably occurred, the SNCt differences appeared almost immediately when the third marker was presented. This may show the process to reduce the processing time. In T1|T2 = 280|200, in which temporal assimilation usually would not occur, the SNCt differences of perceptual equality/inequality started about 40 ms after the third marker. This suggests that the brain activation corresponding to “unequal” perception appeared within 80 ms after the stimuli. Hence, the model is likely to explain the processing of temporal assimilation in the brain. The SNCt continued up to about 400 ms after the end of T2, and was established as a signature of equality/inequality judgment caused by the comparison of the two neighboring time intervals.

Previous literature has reported that the right dorsolateral prefrontal cortex is involved in tasks of cognitive time estimation (Rubia and Smith, 2004), especially in comparison of time intervals (Rao et al., 2001). The SNCt, which is related to the equal/unequal judgments, emerges most prominently around the right-frontal electrodes (i.e., Fp2, F8, and F4). This suggests that the right dorsolateral prefrontal cortex could be a generator of the SNCt (Figures 3, 6). Other imaging techniques, such as magnetoencephalography, should be introduced in order to clarify the spatio-temporal characteristics of this component. Brain activity related to the perceptual equality/inequality of neighboring time intervals thus appeared clearly, and “equal” judgments and “unequal” judgments corresponded to different ERP patterns—for the same stimulus patterns.

### Conflict of interest statement

The authors declare that the research was conducted in the absence of any commercial or financial relationships that could be construed as a potential conflict of interest.

## Footnotes

^1^Because the two experiments were done about 1 year apart, we analyzed the behavioral data from each experiment separately.

^2^We set TWcnv1 and TWcnv2 to 200 ms after the second marker for both Experiments 1 and 2. In the cases of T2 = 80, 120, 160 ms, the difference waves of these three stimulus patterns would not reflect the memory functions of preceding intervals, because these difference waves were assumed to contain the component after the third marker, and they could not be separated from the SNCt.
